# Repair of a Bacterial Small β-Barrel Toxin Pore Depends on Channel Width

**DOI:** 10.1128/mBio.02083-16

**Published:** 2017-02-14

**Authors:** Gisela von Hoven, Amable J. Rivas, Claudia Neukirch, Martina Meyenburg, Qianqian Qin, Sapun Parekh, Nadja Hellmann, Matthias Husmann

**Affiliations:** a Institute of Medical Microbiology and Hygiene, University Medical Center, Johannes Gutenberg University, Mainz, Germany; b Max Planck Institute for Polymer Research, Mainz, Germany; c Institute of Molecular Biophysics, Faculty of Biology, Johannes Gutenberg University Mainz, Mainz, Germany; University of Padova; Max Planck Institute for Infection Biology

## Abstract

Membrane repair emerges as an innate defense protecting target cells against bacterial pore-forming toxins. Here, we report the first paradigm of Ca^2+^-dependent repair following attack by a small β-pore-forming toxin, namely, plasmid-encoded phobalysin of *Photobacterium damselae* subsp. *damselae*. In striking contrast, *Vibrio cholerae* cytolysin, the closest ortholog of phobalysin, subverted repair. Mutational analysis uncovered a role of channel width in toxicity and repair. Thus, the replacement of serine at phobalysin´s presumed channel narrow point with the bulkier tryptophan, the corresponding residue in* Vibrio cholerae* cytolysin (W318), modulated Ca^2+^ influx, lysosomal exocytosis, and membrane repair. And yet, replacing tryptophan (W318) with serine in *Vibrio cholerae* cytolysin enhanced toxicity. The data reveal divergent strategies evolved by two related small β-pore-forming toxins to manipulate target cells: phobalysin leads to fulminant perturbation of ion concentrations, closely followed by Ca^2+^ influx-dependent membrane repair. In contrast, *V. cholerae* cytolysin causes insidious perturbations and escapes control by the cellular wounded membrane repair-like response.

## IMPORTANCE

Previous studies demonstrated that large transmembrane pores, such as those formed by perforin or bacterial toxins of the cholesterol-dependent cytolysin family, trigger rapid, Ca^2+^ influx-dependent repair mechanisms. In contrast, recovery from attack by the small β-pore-forming *Staphylococcus aureus* alpha-toxin or aerolysin is slow in comparison and does not depend on extracellular Ca^2+^. To further elucidate the scope of Ca^2+^ influx-dependent repair and understand its limitations, we compared the cellular responses to phobalysin and *V. cholerae* cytolysin, two related small β-pore-forming toxins which create membrane pores of slightly different sizes. The data indicate that the channel width of a small β-pore-forming toxin is a critical determinant of both primary toxicity and susceptibility to Ca^2+^-dependent repair.

## INTRODUCTION

Pore-forming proteins are widely used by bacteria to directly damage cells (1), promote intracellular growth (2, 3), or introduce virulence factors into the cytosol (4, 5). Intriguingly, nucleated cells are able to restore structural and functional plasma membrane (PM) integrity after damage by bacterial pore-forming toxins (PFTs) (6), permitting, for example, recovery from major *Staphylococcus aureus* alpha-toxin-dependent losses of cellular ATP (7). Restoration of PM integrity has been also documented for streptolysin O (SLO) (8), pneumolysin (9), aerolysin, and listeriolysin (LLO) (10); it occurs in various cell types in culture and has been shown in *Caenorhabditis elegans* to operate *in vivo* (11). Efficient repair of the PM after wounding or attack by proteins forming large pores, such as SLO, perforin, or complement, is thought to require Ca^2+^ influx (8, 12–15). Downstream mechanisms include endocytosis of lesions and replacement of the PM by lysosomal exocytosis (15–22) and/or blebbing of the PM and ectocytosis (23, 24). These pathways might act in a complementary manner (25). Caveolin has been implicated in endocytosis of SLO pores (22). More recently, a requirement of the endosomal complex required for transport (ESCRT) for membrane repair after damage by laser light has been reported; this pathway could also be involved in the repair of membrane pores (26). Notably, the recuperation of cells from an attack by small β-pore-forming *S. aureus* alpha-toxin or aerolysin is significantly slower than that of cells treated with SLO or LLO, and it proceeds in the absence of extracellular Ca^2+^ (10, 27, 28). Furthermore, comparative studies showed that recovery following attack by *S. aureus* alpha-toxin or aerolysin, but not by SLO or LLO, involves p38 mitogen-activated protein kinase (p38 MAPK), autophagy, and phosphorylation of the α-subunit of eukaryotic initiation factor 2 (eIF2α) (10, 28, 29). Evidently, the mode and efficacy of PM repair and cellular recovery depend on the type of PFT (reviewed in reference 5). In order to comprehend differential cellular tolerance for various PFTs and PFT-producing bacteria, it will be important to elucidate the scope and limitations of Ca^2+^ influx-dependent repair. Here, we have investigated cellular responses to phobalysin P (PhlyP) and the orthologous *Vibrio cholerae* cytolysin (VCC) (30–35), two related small β-PFTs of *Photobacterium damselae* subsp. *damselae* and *V. cholerae*, respectively. *V. cholerae* is the notorious cause of a profuse, life-threatening diarrhea in humans. *P. damselae* subsp. *damselae* is a pathogen of marine animals that may infect wounds and lead to hyperaggressive necrotizing soft tissue infection or sepsis in humans. In addition to other proteins (36, 37), VCC and PhlyP are considered to serve as virulence factors of these bacteria (35, 38, 39). We exploited the similar, yet distinct structures of these toxins to gain insight into the function or failure of Ca^2+^ influx-dependent repair after attack by small β-PFTs.

## RESULTS

### PhlyP and VCC perturb ion concentrations in epithelial cells with different kinetics.

**FIG 1:**
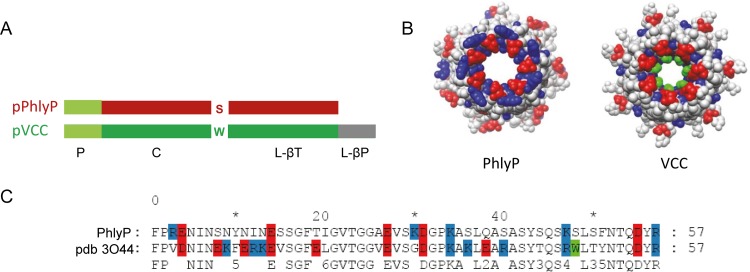
Comparison of phobalysin (PhlyP) and *Vibrio cholerae* cytolysin (VCC). (A) Domain structures of protoxins pPhlyP and pVCC. P, prodomain; C, cytolysin domain; L-βT, β-trefoil lectin domain; L-βP, β-prism lectin domain. (B) Space fill representation of heptameric pore complexes of PhlyP and VCC shown from cytosolic side. Note heptad of tryptophan (green) narrowing the lumen of the VCC pore. (C) Amino acid sequence alignment of channel regions. Color code in panel B is as in panel C. The structure of the presumed PhlyP heptamer was created *in silico* by using MODELLER with the VCC heptamer (30–35) as a template.

**FIG 2:**
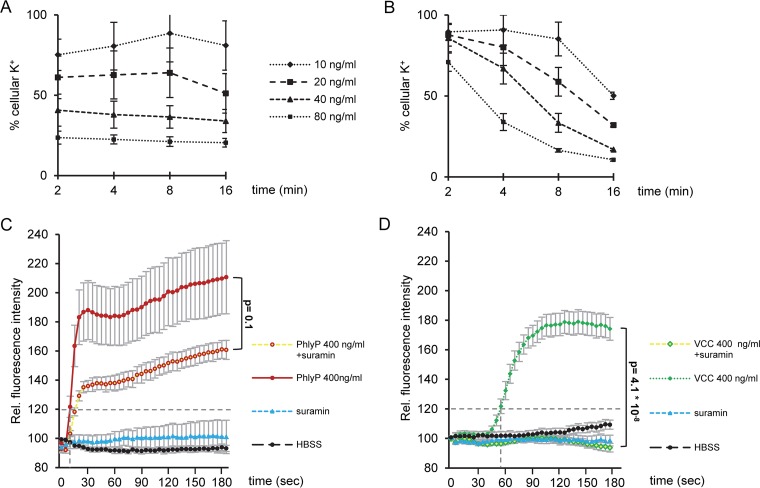
PhlyP and VCC differentially impact cellular K^+^ and Ca^2+^ concentrations. (A, B) Cellular K^+^ was determined by flame photometry with lysates of HaCaT cells obtained after exposure of cells to various concentrations of PhlyP (A) or VCC (B) for the indicated times. Values represent percentages of the results for untreated controls; mean values ± standard errors (SE) are shown (*n* ≥ 3). (C) Cells were pretreated or not with 150 µg/ml suramin, loaded with Fluo-8 AM in the presence or absence of the inhibitor, and exposed to PhlyP (400 ng/ml). Fluorescence intensity was recorded at intervals of 5 s for a period of 3 min; dashed horizontal line indicate threshold used to compare lag times (intersection of *x* axis and dashed perpendicular line). (D) The experiment was performed as described in the legend to panel C, but VCC was examined instead of PhlyP. (C, D) Data are percentages of the results for untreated controls; mean values ± SE are shown (*n* ≥ 4). Statistical significance was determined with Student’s *t* test.

PhlyP is a small β-PFT that is related to VCC (39), but in contrast to VCC, it lacks a C-terminal β-prism domain (Fig. 1A). Moreover, homology-based modeling of the PhlyP transmembrane pore using the known structure of VCC (40) as a scaffold predicted a wider narrow point of the channel (Fig. 1B) and fewer charged residues clustering in the channel-forming region of PhlyP (Fig. 1C). Therefore, in spite of their homology—50% identity on the amino acid level—it is conceivable that PhlyP and VCC exert different effects on target cells. This conjecture was confirmed by the finding that only PhlyP made epithelial cells (HaCaT cells) permeable to propidium iodide (PI) (39), prompting us to also compare changes of ion concentrations in epithelial cells. Loss of intracellular K^+^ is a hallmark of PM permeabilization by all PFTs investigated so far. Treatment of HaCaT cells with purified PhlyP caused dose-dependent loss of cellular K^+^ within 2 min; little further decrease was observed thereafter (Fig. 2A). In contrast, the loss of K^+^ was progressive in samples treated with VCC (Fig. 2B). Given that PhlyP made cells permeable to PI (molecular weight [MW] of 668.4), we surmised that it would also permit influx of Ca^2+^ ions. Cells permeabilized by PhlyP retained the exquisitely Ca^2+^-sensitive probe Fluo-8 AM (MW of ~1,000) (see Fig. S1A in the supplemental material), which was exploited to detect whether the toxins caused changes of intracellular calcium ion concentrations [Ca^2+^]_i_. PhlyP (400 ng/ml) led to a significant increase of fluorescence in Fluo-8 AM-loaded cells within 30 s after exposure (Fig. 2C); half-maximal effects were reached at 100 ng/ml and saturation at ~200 ng/ml (data not shown). VCC at 400 ng/ml led to a final increase of fluorescence like that of PhlyP (Fig. 2D), although 100 ng/ml VCC remained ineffective (data not shown). Conspicuously, the VCC-dependent increase in fluorescence commenced significantly later than the PhlyP-dependent increase (~60 s versus ~12 s; *P* = 9.5 × 10^−8^). This raised the question of whether the two toxins increased [Ca^2+^]_i_ via different mechanisms. Purinoceptors have been implicated in cellular responses to PFT and in the regulation of Ca^2+^ influx (41–43). Therefore, we tested the effect of suramin, an inhibitor of P2 receptors, on PFT-dependent changes of [Ca^2+^]_i_. Suramin exerted a moderate inhibitory effect on the PhlyP-dependent rise of [Ca^2+^]_i_ (Fig. 2C) but virtually blocked the VCC-dependent increase (Fig. 2D).

10.1128/mBio.02083-16.2 FIG S1  PhlyP triggers Ca^2+^ influx and Ca^2+^ influx-dependent recovery of K^+^, and DPA inhibits replenishment of K^+^ after membrane permeabilization by PhlyP. (A) Sequential images from video microscopy of HaCaT cells loaded with Fluo-8 AM and treated with PhlyP at 100 ng/ml. Intensities are represented in false color: red, high intensity; blue, low intensity. (B) HaCaT cells were exposed to 500 ng/ml PhlyP. Cellular K^+^ levels were determined immediately (10 min) or after samples were washed and then incubated for 2 h in the absence of toxin. Values represent percentages of the results for untreated controls; mean values ± SE are shown (*n* = 3). (C) After exposure of HaCaT cells to 150 ng/ml PhlyP for 10 min or after a recovery period of 2 h in the presence or absence of Ca^2+^, cellular K^+^ was determined as described in the legend to panel B. Data are percentages of the results for untreated controls; mean values ± SE are shown (*n* = 4). (D, E) HaCaT cells were preincubated or not with 50 mM desipramine (DPA), 50 mM blebbistatin, or a combination of DPA and blebbistatin. After exposure to 100 ng/ml PhlyP (D) or 5 ng/ml VCC (E) for 10 min or an additional recovery period of 2 h, cellular K^+^ was determined; mean values ± SE from 3 experiments are shown. Download FIG S1, PDF file, 0.1 MB. Copyright © 2017 von Hoven et al. 2017 von Hoven et al. This content is distributed under the terms of the Creative Commons Attribution 4.0 International license.

### Epithelial cells replenish K^+^ after perforation by PhlyP.

**FIG 3:**
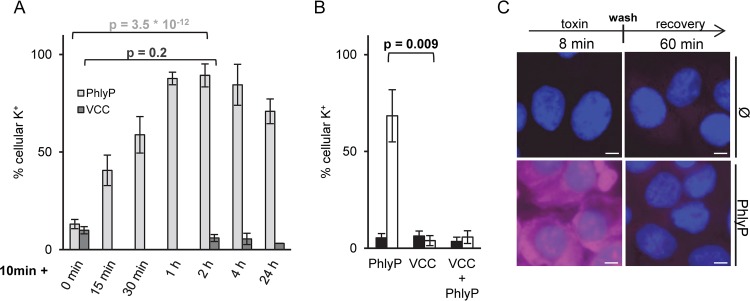
PhlyP, but not VCC, permits rapid reconstitution of membrane integrity. (A) HaCaT cells were incubated with 100 ng/ml PhlyP or VCC for 10 min; K^+^ levels were determined immediately (0 min) or after samples were washed and incubated for various recovery periods, ranging from 15 min to 24 h, in the absence of toxins. Data are percentages of the results for untreated controls; mean values ± SE are shown (*n* ≥ 3). (B) Cells were treated with VCC, PhlyP, or a mixture of both (100 ng/ml each) and incubated for 10 min at 37°C before K^+^ was measured (black bars). Alternatively, the 10-min incubation with toxin(s) was followed by washing out of toxin(s) and subsequent incubation for 2 h in toxin-free medium before K^+^ was measured (white bars). Data shown are mean values ± SE (*n* = 3). (A, B) *P* values were determined with Student’s *t* test. (C) HaCaT cells were treated with mature PhlyP (ECPs from PhlyP-producing *P. damselae* subsp. *damselae* strain AR119) or with ECPs from a nontoxigenic strain (Ø) for 8 min at 37°C. Subsequently, cells were immediately stained for 1 min with PI (50 μg/ml), fixed, and stained with Hoechst 33342 (left) or washed and incubated for a recovery period of 1 h in the absence of ECPs prior to staining (right). Representative images are shown. Scale bar = 10 μm.

To investigate whether PhlyP-treated epithelial cells were able to recover, we measured cellular K^+^ levels immediately after a brief incubation with toxin or after incubation for various times in the absence of toxin. Following incubation of cells with PhlyP (100 ng/ml for 10 min at 37°C), the cellular K^+^ levels were reduced to ~10%, but they returned to normal within 1 h after the removal of unbound toxin (Fig. 3A). A similar recovery was observed when cells were treated with 500 ng/ml PhlyP (see Fig. S1B in the supplemental material). In contrast, the loss of cellular K^+^ in response to VCC was sustained (Fig. 3A). Notably, the combination of both toxins behaved like VCC alone, indicating that the rescue process, apparently triggered by PhlyP, cannot save cells simultaneously intoxicated by VCC (Fig. 3B). Although incubation of cells with PhlyP for 8 min sufficed to cause significant influx of PI, membrane integrity was reconstituted after the washing out of PhlyP. Resealing was observed whether cells were treated with purified PhlyP (data not shown) or extracellular products (ECPs) from strain AR119, a *P. damselae* subsp. *damselae* strain expressing PhlyP (Fig. 3C) (39, 44).

### PhlyP elicits a wounded membrane repair-like response, but VCC does not.

**FIG 4:**
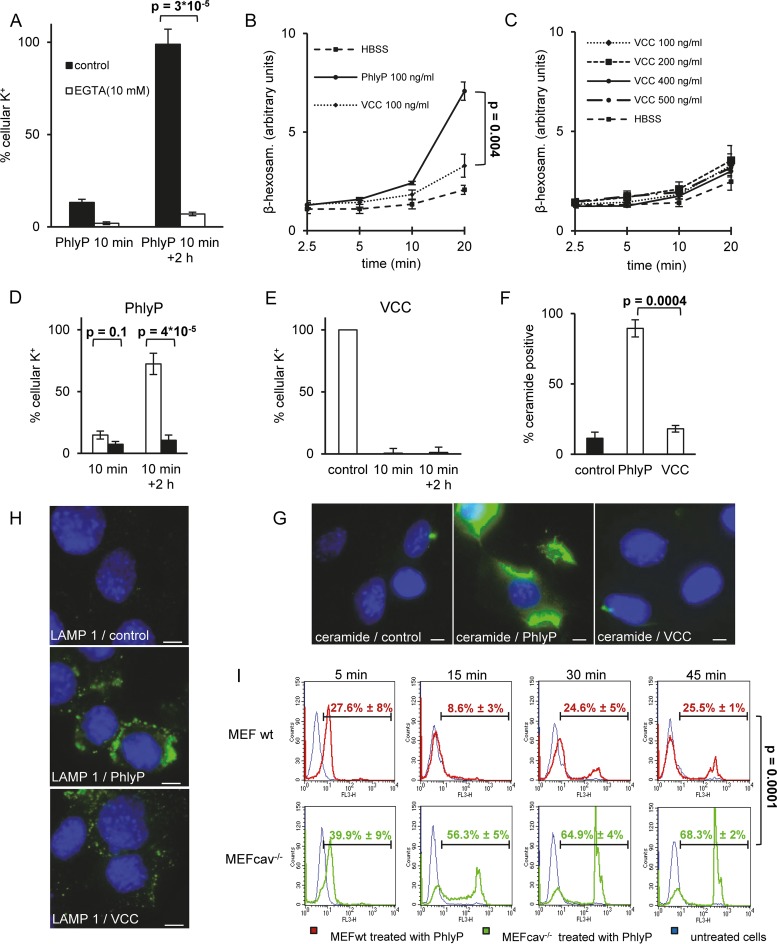
PhlyP triggers a wounded membrane repair-like response. (A) HaCaT cells were incubated with 100 ng/ml PhlyP with or without EGTA (10 mM) for 10 min; K^+^ was determined immediately (10 min) or after washing and incubation for 2 h in the absence of toxin. (B) HaCaT cells were incubated at 37°C with PhlyP or VCC in Hanks balanced salt solution (HBSS) or with HBSS only for various times prior to determination of β-hexosaminidase in supernatants. Mean values ± SE from ≥3 experiments are shown. (C) The experiment was performed as described in the legend to panel B except that various concentrations of VCC were used. (D, E) MEFwt (white bars) or MEFcav^−/−^ (black bars) were treated with PhlyP (D) or VCC (E) at 100 ng/ml. Toxins were present for 10 min before K^+^ was measured in cell lysates. To assess recovery, cells were washed after the 10 min and cultured for another 2 h in the absence of toxin. Mean values ± SE are shown (*n* ≥ 4). (F, G) MEFwt cells were treated or not with PhlyP or VCC at 100 ng/ml for 8 min and surface stained with anticeramide antibody, nuclei were stained with Hoechst, and ceramide-positive cells were counted by immunofluorescence microscopy. (F) Graph summarizing data from 150 cells per condition; mean values ± SE are shown (*n* = 3). (G) Representative images. Scale bar = 10 µm. (H) MEFwt cells were incubated or not with 100 ng/ml PhlyP or VCC for 10 min at 37°C. Subsequently, cells were surface stained for LAMP1 and nuclei were stained with Hoechst. Scale bar = 10 µm. (I) MEFwt or MEFcav^−/−^ were treated or not treated with PhlyP (ECPs of toxigenic strain; 3-µg/ml total protein) for the indicated times, stained with PI for 1 min, and analyzed by flow cytometry. Percentages of PI-positive cells as defined by the cursor are indicated; mean values ± SE are shown (*n* = 3). *P* values in panels A, B, D, F, and I were determined with Student’s *t* test.

Because EGTA prevented the restoration of K^+^ levels (Fig. 4A), and because the depletion of K^+^ was irreversible in Ca^2+^-free medium (see Fig. S1C in the supplemental material), we investigated whether mechanisms proposed to act downstream from Ca^2+^ influx-dependent repair of large membrane pores (15, 18, 22–24) were also involved here. Therefore, we measured the release of β-hexosaminidase, a marker of lysosomal exocytosis (45). Notably, PhlyP causes no leakage of lactate dehydrogenase (39), and EGTA blocked the release of β-hexosaminidase (data not shown), demonstrating that β-hexosaminidase release faithfully reported lysosomal exocytosis. PhlyP induced release of the enzyme from HaCaT cells (Fig. 4B), but VCC was ineffective (Fig. 4B and C). In line with a role of lysosomal exocytosis for recovery from PhlyP attack, desipramine (DPA), an inhibitor of acid sphingomyelinase (ASM), which impairs the reversal of SLO-dependent membrane permeabilization (18), reduced the replenishment of cellular K^+^ without altering the initial toxin-dependent loss of this ion (see Fig. S1D). Consistent with this, inhibition of ASM did not aggravate the VCC-dependent loss of K^+^ (see Fig. S1E). PhlyP caused the formation of large, dynamic blebs in HaCaT cells, some of which appeared to detach to form large vesicles (see Movie S1), and blebbistatin stalled the formation of free vesicles (see Movie S2). However, neither alone nor in combination with DPA did blebbistatin alter the replenishment of cellular K^+^ after attack by PhlyP (see Fig. S1D), thus not supporting a major role of blebbing for cellular recovery from PhlyP. And yet, PhlyP led to increased endocytosis of fluorescently labeled bovine serum albumin (BSA), a cargo of caveolar uptake (see Fig. S2A), and SDS-stable oligomers were coisolated with caveolin and the exosomal marker protein flotillin in supernatants of target cells, suggesting sequential endocytosis and exocytosis of PhlyP (see Fig. S2B). The wounded membrane repair-like response following membrane perforation by SLO has been demonstrated by RNA interference (RNAi) to depend on caveolin (22), and a current model is depicted in Fig. S2D. Here, we exploited mouse embryonal fibroblasts (MEF) lacking caveolin expression (MEFcav^−/−^) to investigate whether this protein is also important for cellular defense against the small β-pore-forming toxin PhlyP. As in HaCaT cells, VCC and PhlyP both caused loss of K^+^ from wild-type MEF (MEFwt) or MEFcav^−/−^ (Fig. 4D and E). Also, restoration of the intracellular K^+^ concentration was not observed after treatment with VCC (Fig. 4E), whereas replenishment of K^+ ^was efficient in PhlyP-treated wild-type cells. Importantly, replenishment of K^+ ^failed in MEFcav^−/−^ exposed to PhlyP (Fig. 4D). That PhlyP triggered a wounded membrane repair-like response in MEF was further suggested by the facts that it increased the fraction of cells carrying ceramide (Fig. 4F and G) and that it led to exposure of a luminal epitope of the lysosomal marker protein LAMP-1 at the cell surface (Fig. 4H) (17); only minor effects were discernible after treatment with VCC. The protective role of caveolin was confirmed when we analyzed PI influx by flow cytometry. Exposure to PhlyP for 5 min led to some influx of PI in wild-type and caveolin-deficient cells. However, wild-type cells soon excluded PI again (15 min), whereas the fraction of MEFcav^−/−^ positive for PI had increased (Fig. 4I). By 45 min, two-thirds of MEFcav^−/−^ but only about one-fourth of wild-type cells were heavily stained with PI.

10.1128/mBio.02083-16.3 FIG S2  PhlyP induces endocytosis, and PhlyP oligomers are released by target cells. (A) HaCaT cells were incubated with PhlyP at 75 ng/ml at 37°C for 15 min in the presence of BSA-Alexa Fluor 488 (50 μg/ml) before being processed for analysis by fluorescence microscopy. Representative images out of >10 are shown. (B) For quantification, HaCaT cells were treated as described in the legend to panel A, incubated for various times, and analyzed by flow cytometry. Mean values ± SE are shown (*n* = 3). (C) HaCaT cells were preincubated with dimethyl sulfoxide (DMSO) or 50 µM blebbistatin in DMSO for 30 min at 37°C, loaded with 100 ng/ml PhlyP, washed, and incubated for 1 h at 37°C. Supernatants were collected, and pellets from sequential centrifugation steps were analyzed by Western blotting for the presence of PhlyP, flotillin, and caveolin-1. (D) A current model of repair of large pores formed by SLO. SLO pores cause rapid Ca^2+^ influx, triggering lysosomal exocytosis and release of acidic sphingomyelinase (ASM). The enzyme catalyzes transformation of sphingomyelin to ceramide, leading to enhanced caveolar endocytosis of membrane with lesions (22). Download FIG S2, PDF file, 0.4 MB. Copyright © 2017 von Hoven et al. 2017 von Hoven et al. This content is distributed under the terms of the Creative Commons Attribution 4.0 International license.

10.1128/mBio.02083-16.9 MOVIE S1  PhlyP triggers increase of intracellular Ca^2+^, blebbing of the PM, and formation of vesicles. HaCaT cells were loaded with Fluo-8 AM and incubated at 37°C using a heated microscope stage. Fluorescence video microscopy was performed. First image in sequence is of untreated cells; subsequent images were taken every 5 s following injection of purified PhlyP (100 ng/ml). Download MOVIE S1, MOV file, 1.7 MB. Copyright © 2017 von Hoven et al. 2017 von Hoven et al. This content is distributed under the terms of the Creative Commons Attribution 4.0 International license.

10.1128/mBio.02083-16.10 MOVIE S2  Blebbistatin stalls PhlyP-dependent vesicle formation. HaCaT cells were preincubated for 30 min with blebbistatin (50 μM), loaded with Fluo-8 AM, and incubated at 37°C on a heated microscope stage. Fluorescence video microscopy was performed. First image in sequence is of cells without toxin; subsequent images were taken every 5 s following injection of PhlyP (ECPs from strain AR119 cells, 60 μg/ml total protein, corresponding to ~1 μg/ml PhlyP). Download MOVIE S2, MOV file, 1.5 MB. Copyright © 2017 von Hoven et al. 2017 von Hoven et al. This content is distributed under the terms of the Creative Commons Attribution 4.0 International license.

### Recovery from PhlyP is not blocked by the p38 MAPK inhibitor SB203580.

In contrast to PhlyP, other small β-pore-forming toxins, i.e., aerolysin and *S. aureus* alpha-toxin, have been previously shown to trigger not Ca^2+^ influx-dependent repair but slower, p38 MAPK-dependent recovery processes (28). Like VCC, PhlyP activates p38 MAPK (see Fig. S3A in the supplemental material). However, an inhibitor of activated p38 MAPK, SB203580, did not impede the replenishment of cellular K^+^ in PhlyP-treated cells (see Fig. S3B).

10.1128/mBio.02083-16.4 FIG S3  p38 MAPK is activated by PhlyP but dispensable for recovery. (A) HaCaT cells were treated with 100 ng/ml PhlyP or VCC for the indicated times. Whole-cell lysates were analyzed by Western blotting for (P)-p38 (phosphorylated p38) and p38 as indicated. (B) HaCaT cells were pretreated for 1 h with SB203580 (20 μM) or solvent alone (control) before PhlyP was added to a final concentration of 100 ng/ml. Samples were incubated for 10 min in the continuous presence of solvent/inhibitor. Subsequently, samples were either lysed immediately or washed and incubated in the absence of solvent/inhibitor for 2 h (recovery) before they were lysed and K^+^ was measured. Values represent percentages of the results for untreated controls. Mean values ± SE are shown (*n* = 3). (B) *P* value was determined with Student’s *t* test. Download FIG S3, PDF file, 0.2 MB. Copyright © 2017 von Hoven et al. 2017 von Hoven et al. This content is distributed under the terms of the Creative Commons Attribution 4.0 International license.

### Channel narrow points impact fluxes of Ca^2+^.

**FIG 5:**
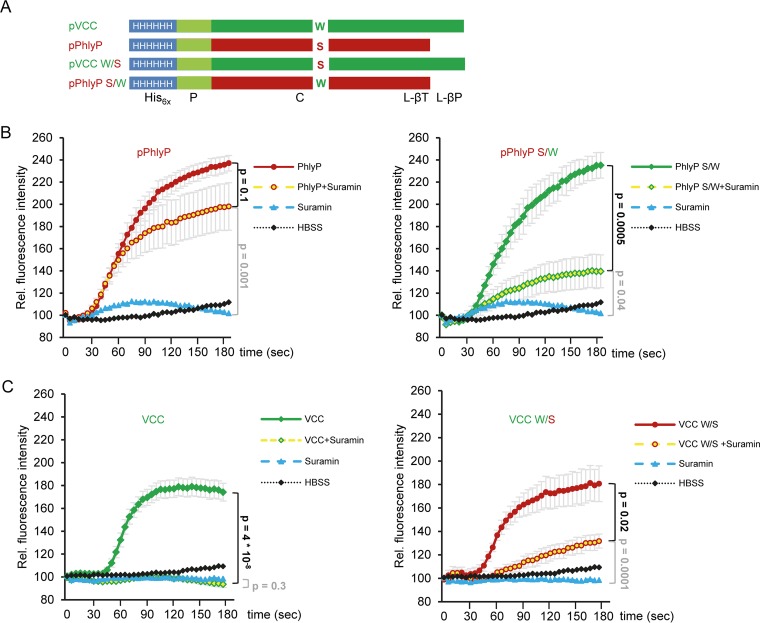
Channel narrow points codetermine toxin-dependent changes of intracellular [Ca^2+^]. (A) Schematic representation of His_6_-tagged pPhlyP, pPhlyP S/W mutant, pVCC, and pVCC W/S mutant; domain designations are as defined in the legend to Fig. 1A. Colored letters in cytolysin domain indicate residues exchanged in mutants. (B and C) HaCaT cells were pretreated or not with 150 µg/ml suramin, loaded with Fluo-8 AM in the presence or absence of inhibitor, and exposed to pPhlyP or pPhlyP S/W (B) or to VCC or VCC W/S (C) at 400 ng/ml. Fluorescence intensity was recorded at intervals of 5 s for a period of 3 min. Mean values ± SE are shown (*n* ≥ 5). *P* values were determined with Student’s *t* test.

The differential effects of PhlyP versus VCC on cellular homeostasis, influx of vital dyes, and repair could be due at least in part to differences in their transmembrane channels. Specifically, the bulky side chain of W318 in the VCC channel forms a heptad, reminiscent of the phenylalanine clamp in the anthrax protective antigen pore (4, 40), and it could restrict the flux of ions or dyes. In contrast, serine 341, predicted to form the narrow point in PhlyP pores, is expected to be less obstructive. To investigate a potential impact of channel narrow points on toxin function, we generated single-amino-acid exchange mutants of the VCC and PhlyP protoxins pVCC and pPhlyP, in which tryptophan at position 318 (W318) of pVCC and serine at position 341 of pPhlyP were swapped, creating mutants pVCC(W318S) and pPhlyP(S341W) (Fig. 5A). HaCaT cells were loaded with Fluo-8 AM and treated with wild-type or mutant toxins, and fluorescence was recorded; the experiment was performed in the presence or absence of suramin. Intriguingly, pPhlyP(S341W)—hereinafter termed pPhlyP S/W—caused a significantly lower suramin-insensitive increase of fluorescence than pPhlyP (Fig. 5B). For comparison of wild-type and mutant VCC (Fig. 5C), the mature toxins were generated with trypsin, because maturation by cellular proteases appeared comparably inefficient; VCC(W318S)—termed VCC W/S below—caused increases of fluorescence similar to those caused by wild-type VCC, but only in the case of VCC W/S was a significant portion of that signal insensitive to suramin (Fig. 5C).

### Narrowing the PhlyP channel limits primary damage and repair; widening the VCC channel enhances toxicity.

**FIG 6:**
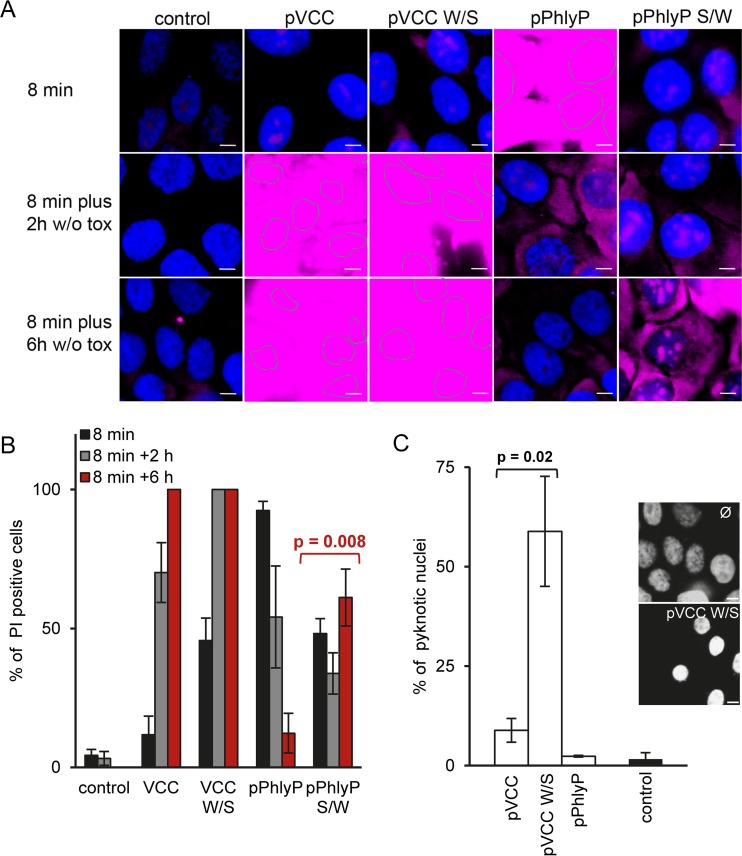
Point mutations in toxin channels modulate toxicity and reparability. (A) HaCaT cells were incubated with protoxins (100 ng/ml). After 8 min, cells were washed and either stained immediately with PI for 1 min or incubated for recovery periods of 2 or 6 h before incubation with dye; cells were then fixed and processed for microscopic analysis. To reveal weak influx of PI in some samples and to apply equal exposure to all images, some images had to be overexposed; nuclei in overexposed samples are indicated by dotted lines. For corresponding single-channel grey-scale images (lower exposure), see Fig. S4 in the supplemental material. (B) Graph summarizes percentages of PI-positive cells. A total of ≥150 cells were evaluated per condition; mean values ± SE are shown (*n* ≥ 4). (C) Graph shows percentages of pyknotic nuclei (condensed and intensely stained with Hoechst) for samples treated as described in the legend to panel B. Mean values ± SE are shown (*n* = 3). Representative images of pVCC W/S-treated cells and untreated control cells are displayed. Scale bar = 20 µm. *P* values were determined with Student’s *t* test.

**FIG 7:**
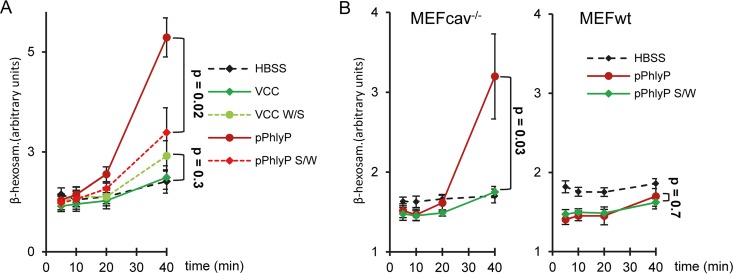
Single-amino-acid exchange at the narrow point of the PhlyP channel reduces capacity to trigger lysosomal exocytosis. (A) HaCaT cells were incubated with indicated (pro)toxins (100 ng/ml) for various times before β-hexosaminidase was determined in supernatants. (B) MEFwt or MEFcav^−/−^ were incubated with pPhlyP or pPhlyP S/W (100 ng/ml) for various times before β-hexosaminidase was determined in supernatants. Mean values ± SE are shown (*n* = 4). *P* values were determined with Student’s *t* test.

Next, we asked whether the above-described point mutations had an impact on membrane damage or repair. Cells were treated with wild-type or mutant protoxins, stained with PI and Hoechst stain, and examined by fluorescence microscopy. Only minimal influx of PI was noted upon a short incubation with either wild-type or mutant pVCC, but loss of membrane integrity progressed inexorably despite washing out of toxin (Fig. 6A and B; see also Fig. S4 in the supplemental material). Notably, cells deteriorated more rapidly after exposure to pVCC W/S, as suggested by particularly strong influx of PI and nuclear condensation (see Fig. S4 and 6B and C, respectively). The swapping of serine and tryptophan in PhlyP led to more pronounced changes: only in half of the cells was influx of PI observed, and this only to a small extent, upon treatment with pPhlyP S/W, while the majority of cells treated with wild-type pPhlyP were brightly stained. Surprisingly, pPhlyP S/W-treated cells continued to permit low-level ingress of the dye, while membrane integrity was restored in pPhlyP-treated cells. Quantification of SDS-stable oligomers and analysis by electron microscopy did not indicate alterations in the ability to form oligomers (see Fig. S5). Individual PhlyP pores were of equal or slightly higher conductance than mutant pores for all salts tested, but the traces for the PhlyP S/W mutant pores showed increased levels of flickering and frequent breakdown of conductance, which lasted for seconds in some cases (see Fig. S6). We also found that PhlyP pores (mutant or wild type) showed higher conductance than VCC pores (about 300 versus 22 pS in KCl) and were rather cation selective, while VCC was moderately anion selective (46). Differential behavior of pPhlyP and pPhlyP S/W was also evident in MEF cells. First, the influx of PI was more pronounced in response to pPhlyP (see Fig. S7A). Second, pPhlyP caused a far stronger release of β-hexosaminidase in HaCaT cells (Fig. 7A) or caveolin-deficient MEF (Fig. 7B) than did pPhlyP S/W. This release was completely blocked by EGTA (data not shown). The binding of pPhlyP to wild-type or knockout MEF was equal (Fig. S7B), but MEFcav^−/−^ cells were more sensitive to pPhlyP (Fig. S7A). The difference between PhlyP and pPhlyP S/W was more pronounced in wild-type cells (Fig. S7A). Similarly, DPA sensitized MEFwt for both PhlyP and pPhlyP S/W (see Fig. S7C). Thus, late steps of the wounded membrane repair-like response (ASM- and caveolin-dependent steps; see Fig. S2D) appear to be involved in ongoing defense of MEF against PhlyP or pPhlyP S/W. The different capacities of PhlyP and pPhlyP S/W to trigger the release of β-hexosaminidase surfaced when caveolin-dependent tolerance was disabled (Fig. 7B).

10.1128/mBio.02083-16.5 FIG S4  Single-channel grey-scale images corresponding to the images in Fig. 6A. HaCaT cells were incubated with protoxins (100 ng/ml). After 8 min, cells were washed and either immediately incubated with 50 µg/ml PI for 1 min at room temperature or incubated for 2 h or 6 h before incubation with PI, fixation, and microscopic examination. Download FIG S4, PDF file, 0.9 MB. Copyright © 2017 von Hoven et al. 2017 von Hoven et al. This content is distributed under the terms of the Creative Commons Attribution 4.0 International license.

10.1128/mBio.02083-16.6 FIG S5  pPhlyP S/W binds, oligomerizes, and forms annular structures. (A) Rabbit erythrocyte ghosts were loaded with pPhlyP, pPhlyP S/W, or pVCC and analyzed by transmission electron microscopy. Scale bar = 50 nm. (B) Erythrocyte ghosts were incubated with pVCC, pPhlyP, or pPhlyP S/W and separated by SDS-PAGE, and proteins were silver stained. The filled triangles indicate the presumed oligomers, the open triangles the monomers. Download FIG S5, PDF file, 0.6 MB. Copyright © 2017 von Hoven et al. 2017 von Hoven et al. This content is distributed under the terms of the Creative Commons Attribution 4.0 International license.

10.1128/mBio.02083-16.7 FIG S6  PhlyP pores are of rather high conductance and moderately cation selective; the conductance of PhlyP S/W pores is unstable. (A) Single channel recordings on BLM [black lipid membrane(s)] were obtained with PhlyP or PhlyP S/W. Samples of typical traces are shown. Data were obtained with 100 mM KAc; overlay of raw data after filtering (15-point, 2nd-order Savitzky-Golay filter) is presented. Note well-defined steps with only rare downward spikes with wild-type PhlyP. In contrast, significant downward spiking and periods of reduced conductance were observable with PhlyP S/W; increasing toxin concentration led to rapid increase of the current to levels where individual steps could no longer be identified. (B) Multiple current traces from experiments similar to the ones whose results are shown in panel A were analyzed, and the results are presented as histograms. Left, data for PhlyP; right, PhlyP S/W. White bars represent data obtained with 100 mM LiCl, black bars with 100 mM KAc, and grey bars with 100 mM KCl. The distributions were analyzed based on one or two Gaussian functions. The relative frequency was obtained by normalizing to the total number of steps. Download FIG S6, PDF file, 0.2 MB. Copyright © 2017 von Hoven et al. 2017 von Hoven et al. This content is distributed under the terms of the Creative Commons Attribution 4.0 International license.

10.1128/mBio.02083-16.8 FIG S7  Differential response of MEF to pPhlyP and pPhlyP S/W. (A) MEFwt or MEFcav^−/−^ cells were incubated with pPhlyP or pPhlyP S/W (100 ng/ml) for indicated times, stained with PI for 1 min (50 µg/ml), and analyzed by flow cytometry. (Left) Overlays of histograms; events embraced by the cursor are indicated as percentages of total events. Mean values ± SE are shown (*n* ≥ 3). (Right) Graphs comparing percentages of PI-positive cells in samples treated for 45 min with pPhlyP or pPhlyP S/W. Mean values ± SE are shown (*n* = 8). (B) MEFwt or MEFcav^−/−^ cells were pretreated with TAPI (TNF-alpha protease inhibitor) and subsequently incubated with pVCC or pPhlyP (5 μg/ml) on ice; cells were washed, fixed, and immunostained with anti-His tag antibody and Alexa Fluor 488-labeled secondary antibody for detection of surface-bound protoxins by fluorescence-activated cell sorting (FACS). (C) MEFwt cells were treated and analyzed as described in the legend to panel A in the presence or absence of 30 μM desipramine (DPA). Numbers indicate events under the cursor; mean values ± SE are shown (*n* = 5). (A, C) Statistical significance was determined with Student’s *t* test. Download FIG S7, PDF file, 0.1 MB. Copyright © 2017 von Hoven et al. 2017 von Hoven et al. This content is distributed under the terms of the Creative Commons Attribution 4.0 International license.

## DISCUSSION

The present work reveals that different small β-pore-forming toxins may either trigger or subvert calcium influx-dependent repair. Furthermore, the data suggest that the channel width of small β-pores codetermines the kinetics and degree of primary damage, as well as susceptibility to repair. PhlyP, in contrast to the closely related VCC, caused fulminant breakdown of membrane integrity but permitted resealing by a process which until now has only been implicated in the repair of much larger membrane lesions, for instance, pores formed by cholesterol-dependent cytolysins. The modeling-based hypothesis that the narrow point in the PhlyP channel is wider than that in VCC is supported by conductance measurements.

**FIG 8:**
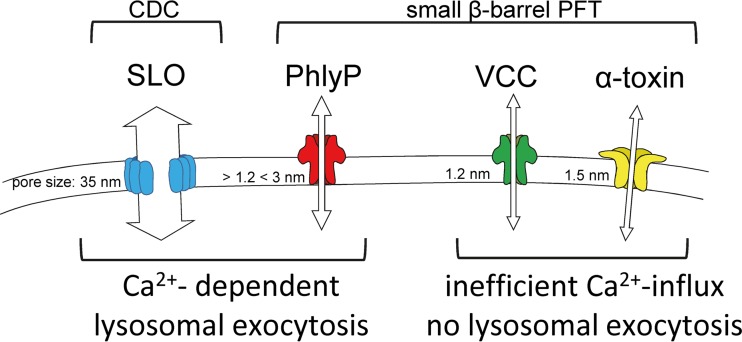
Depending on pore size, small β-PFTs may either trigger or subvert Ca^2+^ influx-dependent repair. PhlyP, a small β-barrel pore-forming toxin which forms comparatively wide pores, triggers rapid Ca^2+^ influx, lysosomal exocytosis, and repair similarly to the large pore-forming streptolysin O (SLO). In contrast, small β-barrel pore-forming toxins like *Vibrio cholerae* cytolysin (VCC) form narrower channels and subvert this response. CDC, cholesterol-dependent cytolysins.

Repair of PhlyP pores involves Ca^2+^ influx, lysosomal exocytosis, and caveolin, but MAPK p38 is dispensable, supporting the idea that Ca^2+^ influx-dependent repair supersedes the requirement for alternative salvage pathways. In striking contrast to PhlyP, VCC subverts Ca^2+^ influx-dependent repair. Thus, even brief exposure of cells to low concentrations of VCC sufficed to initiate the progressive demise of human epithelial cells. That nanomolar concentrations of VCC are required to increase [Ca^2+^]_i_, although picomolar concentrations are sufficient to kill cells (34), provides an explanation for VCC’s propensity to subvert repair. However, even concentrations of VCC sufficient to increase [Ca^2+^]_i_ did not elicit a membrane repair response. Inappropriate topology, timing, or the degree of VCC-dependent increases of [Ca^2+^]_i_ may be responsible: that VCC-dependent increases of [Ca^2+^]_i_ were blocked by suramin indicated that they are mediated by P2 receptors, G-protein-coupled receptors, or other targets of the drug, which might cause Ca^2+^ fluxes not to occur in sufficient proximity to VCC pores to allow repair. Second, compared to the PhlyP-dependent Ca^2+^ influx, the VCC-dependent Ca^2+^ influx was delayed and comparatively slight. Therefore, we believe that the progressive damage by VCC is due to inadequate Ca^2+^ influx through the small and anion-selective pore (34, 40), in the face of otherwise severe perturbations of cellular physiology (e.g., loss of K^+^). Sure enough, VCC proved to be an inefficient trigger of lysosomal exocytosis. Notably, the PhlyP-dependent responses were unable to compensate for VCC’s inability to trigger repair in mixing experiments. This could happen if VCC inhibits a step of the repair program downstream from lysosomal exocytosis, for instance, caveolar endocytosis. Because the abilities of VCC and PhlyP to trigger or subvert Ca^2+^ influx-dependent repair appeared to correlate with their different channel narrow points, mutational analysis was a sensible approach. Single residues presumed to form channel narrow points of PhlyP and VCC were swapped to reveal their contributions to functional phenotypes. Changes in the Ca^2+^ influx resulting from mutations of channel narrow points might impact both the cytotoxic power and ability to trigger repair responses; it was not predictable which effect would prevail. And yet, the interpretation of the data obtained with these constructs was straightforward: W318 restricts the influx of calcium ions through VCC pores; the obstacle falls away in the W318S mutant. Conversely, the replacement of S341 in pPhlyP with tryptophan reduces the influx of calcium ions. A principle finding made with the mutant protoxins was that the effect of channel width on cellular responses depends on the molecular context. The wider narrow point of the PhlyP channel promotes lysosomal exocytosis and recovery. However, it fails to do so if transplanted to VCC; in fact, it enhances toxicity in that context. The reason could be that the moderately increased influx of Ca^2+^ through mutant VCC pores is sufficient to enhance toxicity but too low to trigger Ca^2+^ influx-dependent repair. As a matter of fact, pVCC W/S caused only slightly greater increases of Ca^2+^ influx than did wild-type pVCC, and the increase in the β-hexosaminidase release was statistically insignificant. That pVCC W/S did not elicit a stronger suramin-insensitive increase of [Ca^2+^]_i_ could be due to additional restraints in VCC pores. To sum up, VCC and PhlyP, two related small β-PFTs, pose quite different challenges to cell autonomous defense, which may, at least in part, be attributed to different channel widths (Fig. 8): whereas PhlyP acts fast and thus could overrun host responses if present in sufficient quantities, VCC causes insidious damage and subverts membrane repair. The results highlight the function or failure of Ca^2+^ influx-dependent repair as a defense against small β-PFTs; this may help to better understand the pathogenesis of diseases caused by bacteria producing these widespread toxins.

## MATERIALS AND METHODS

### Toxins.

The preparation of PhlyP and VCC was as described previously (39). In brief, PhlyP was purified by preparative isoelectric focusing and ion exchange chromatography from extracellular products of *P. damselae* subsp. *damselae*. Recombinant protoxins pVCC, pVCC W/S, pPhlyP, and pPhlyP S/W were expressed in *Escherichia coli* as N-terminally His_6_-tagged fusion proteins and purified by affinity chromatography; VCC was generated from pVCC with trypsin (33). Single-amino-acid-exchange mutants of pPhlyP and pVCC were generated with the aid of the QuikChange II XL site-directed mutagenesis kit (Agilent Technologies). For primer sequences and technical details, see Text S1 in the supplemental material.

10.1128/mBio.02083-16.1 TEXT S1  Supplemental materials and methods. Download TEXT S1, DOCX file, 0.04 MB. Copyright © 2017 von Hoven et al. 2017 von Hoven et al. This content is distributed under the terms of the Creative Commons Attribution 4.0 International license.

### *In silico* modeling of the PhlyP pore.

The sequence of PhlyP was modeled on the X-ray structure of VCC (PDB identifier [ID] 3O44 [40]). Alignment and structural modeling were performed by using MODELLER version 9.13 (47). See Text S1 in the supplemental material for details.

### Cells and culture conditions.

HaCaT cells (nonvirally transformed human keratinocytes) (48), wild-type mouse embryonal fibroblasts (MEFwt), or MEFcav^−/−^ (kindly provided by Mario Schelhaas) were cultured in Dulbecco modified Eagle medium (DMEM)–F-12 GlutaMAX-I medium with 10% fetal calf serum, 1% HEPES buffer, 1% penicillin–streptomycin in a humidified incubator with 5% CO_2_ at 37°C. All media and additives were obtained from Life Technologies, Inc.

### Flame photometry for measurement of K^+^.

The loss and replenishment of cellular K^+^ levels after an initial loss is a valuable proxy of perturbation and reconstitution of membrane integrity after attack by small β-PFTs and other PFTs (10, 28). Cellular K^+^ was quantified by flame photometry as described previously (10). In brief, cells were washed three times with ice-cold K^+^-free choline buffer. Cells were subsequently lysed by incubation for 30 min in choline buffer–0.5% Triton X-100 at room temperature on a shaker. Lysates were analyzed for K^+ ^with an M401 flame photometer (Sherwood, United Kingdom) using propane gas.

### Fluo-8 AM-based Ca^2+^ assay.

PFT-induced changes of [Ca^2+^]_i_ in HaCaT cells were monitored by using Fluo-8 AM from Santa Cruz Biotechnology, Inc., in a TriStar LB 941 instrument from Berthold Technologies, as detailed in Text S1 in the supplemental material.

### β-Hexosaminidase release assay.

The β-hexosaminidase release assay was performed as described previously (45).

### Fluorescence microscopy.

Immunofluorescence analysis of ceramide and the lysosomal marker protein LAMP-1 was performed with MEF because the available antibodies yielded unspecific staining in HaCaT cells. The staining protocols for LAMP-1 and ceramide were as described in the supplemental material.

### PI influx.

The PI influx assay was performed as described previously (39). See Text S1 in the supplemental material for details.

### Statistics.

The data shown are from ≥3 independent experiments if not otherwise stated. Error bars represent plus-or-minus standard errors of the means. The statistical significance of differences between mean values was assessed with the two-sided Student’s *t* test or with one-way analysis of variance (ANOVA) for multiple comparison; significance was assumed when the *P* value was ≤0.05.
