# Supplementary material for: Repair of a Bacterial Small β-Barrel Toxin Pore Depends on Channel Width
Source: mBio. 2017 Feb 14;8(1):e02083-16. doi: 10.1128/mBio.02083-16 (PMC5312083; doi:10.1128/mBio.02083-16)
Supplement: FIG S1 [file mbo001173189sf1.pdf]

Figure S1

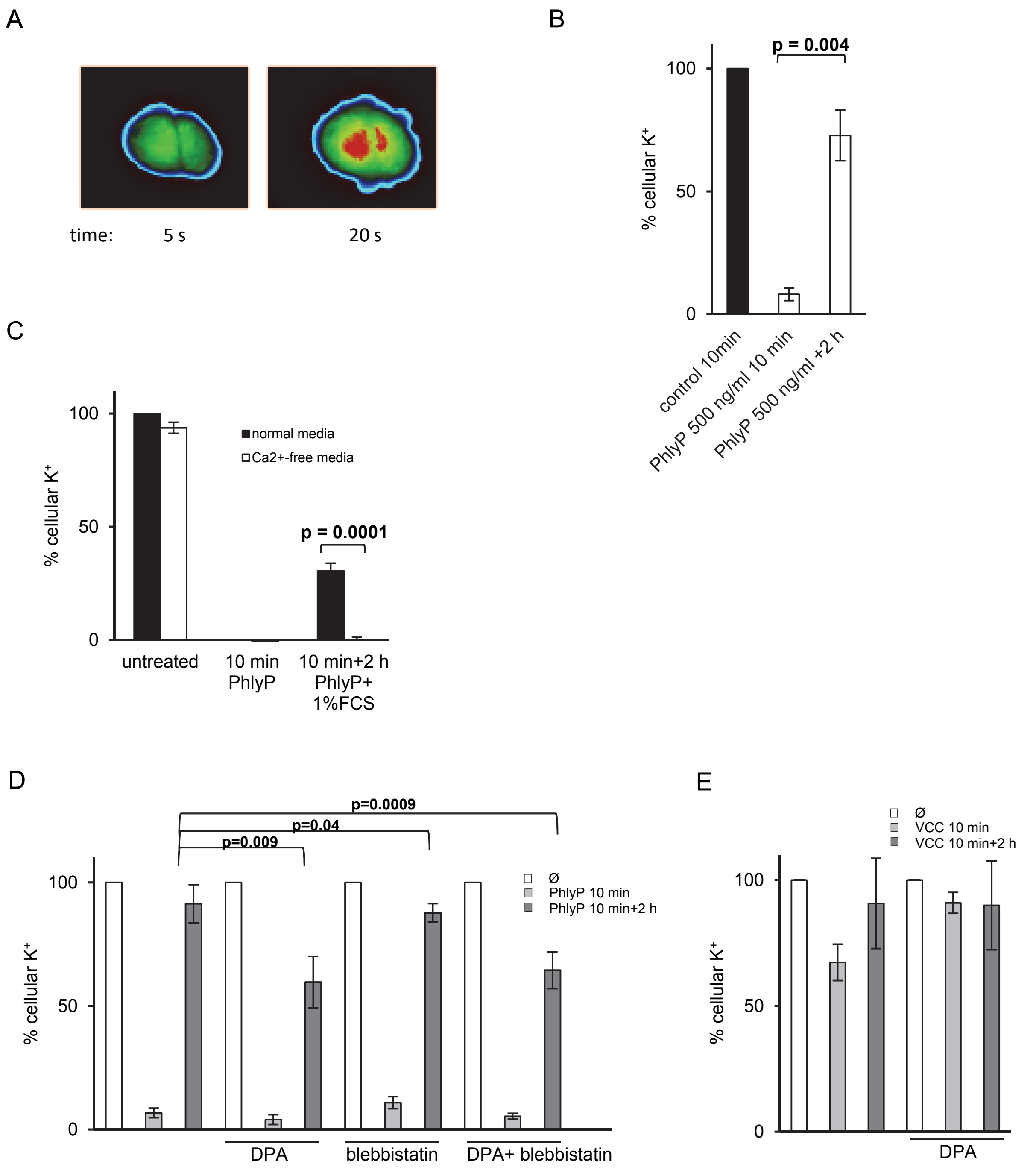

FIG S1 PhlyP triggers Ca^{2+}-influx and Ca^{2+}-influx-dependent recovery of K^+ and DPA inhibits replenishment of K+ after membrane permeabilization by PhlyP. (A) Sequential images from video microscpy of HaCaT cells loaded with Fluo-8AM and treated with PhlyP 100 ng/ml. False-color representation of intensities: red high intensity, blue low intensity. (B) HaCaT cells were exposed to 500 ng/ml PhlyP. Cellular K^+ levels were determined immediately ("10 min"), or after samples were washed and incubated for 2 h, in the absence of toxin. Values represent percent of untreated controls; mean values ± SE; n = 3; (C) After exposure of HaCaT cells to 150 ng/ml PhlyP for 10 min or after a recovery period of 2 h in the presence or absence of Ca^{2+} cellular K^+ was determined as in (B). Data are percent of untreated controls; mean values ± SE; n = 4. (D) HaCaT cells were preincubated, or not with 50 µM desipramine (DPA), 50 µM blebbistatin or the combination of DPA and blebbistatin. After exposure to 100 ng/ml PhlyP or (E) 5 ng/ml VCC for 10 min or a additional recovery period of 2 h cellular K^+ was determined; mean values ± SE of 3 experiments.
