# Supplementary material for: Repair of a Bacterial Small β-Barrel Toxin Pore Depends on Channel Width
Source: mBio. 2017 Feb 14;8(1):e02083-16. doi: 10.1128/mBio.02083-16 (PMC5312083; doi:10.1128/mBio.02083-16)
Supplement: FIG S2 [file mbo001173189sf2.pdf]

Figure S2

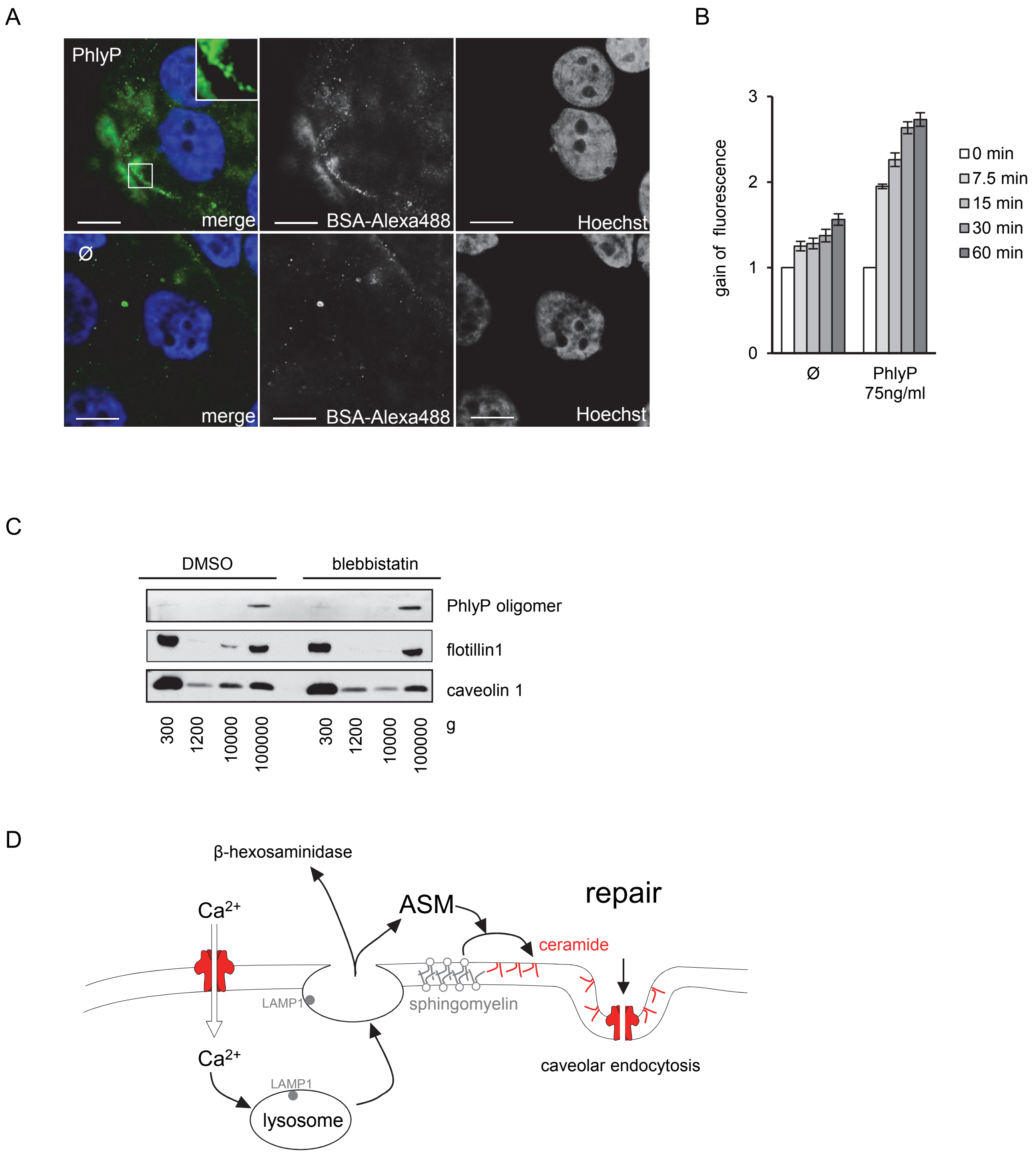

FIG S2 PhlyP induces endocytosis**;** PhlyP oligomers are released by target cells. (A) HaCaT cells were incubated with PhlyP 75 ng/ml at 37°C for 15 min in the presence of BSA-Alexa 488 (50 µg/ml) before being processed for analysis by fluorescence microscopy; representative images out of >10. B) For quantification, HaCaT cells were treated as in (A), incubated for various times and analyzed by flow cytometry. Mean values ±SE; n=3. (C) HaCaT cells were preincubated with DMSO or 50 µM blebbistatin in DMSO (30 min, 37°C), loaded with 100 ng/ml PhlyP, washed and incubated for 1 h at 37°C. Supernatants were collected and pellets from sequential centrifugation steps were analyzed by Western blot for the presence of PhlyP, flotillin and caveolin-1. (D) A current model of repair of large pores formed by SLO. SLO-pores cause rapid Ca^{2+}- influx, triggering lysosomal exocytosis and release of acidic sphingomyelinase (ASM). The enzyme catalyzes transformation of sphingomyelin to ceramide leading to enhanced caveolar endocytosis of lesioned membrane (22).
