# Supplementary material for: Repair of a Bacterial Small β-Barrel Toxin Pore Depends on Channel Width
Source: mBio. 2017 Feb 14;8(1):e02083-16. doi: 10.1128/mBio.02083-16 (PMC5312083; doi:10.1128/mBio.02083-16)
Supplement: FIG S3 [file mbo001173189sf3.pdf]

Figure S3

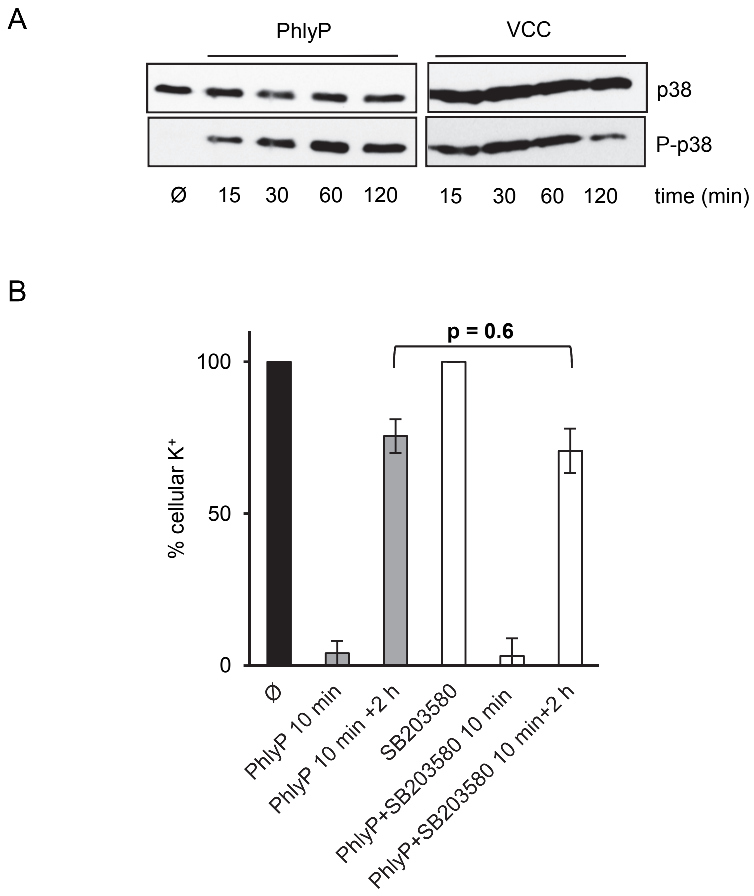

FIG S3 p38 MAPK is activated by PhlyP, but dispensable for recovery. (A) HaCaT cells were treated with 100 ng/ml PhlyP or VCC for the indicated times. Whole cell lysates were analyzed by Western-blot for (P)-p38 and p38 as indicated. (B) HaCaT cells were pretreated for 1 h with SB203580 (20 mM) or solvent alone (control) before PhlyP was added to a final concentration of 100 ng/ml. Samples were incubated for 10 min in the continuous presence of solvent / inhibitor. Subsequently, samples were either lyzed immediately, or washed, and incubated in the absence of solvent / inhibitor for 2 h ("recovery"), before they were lyzed and K^+ was measured. Values represent percent of untreated controls. Data show mean values ± SE; n= 3. The p-value in (B) was determined with Student´s t-test.
