# Supplementary material for: Repair of a Bacterial Small β-Barrel Toxin Pore Depends on Channel Width
Source: mBio. 2017 Feb 14;8(1):e02083-16. doi: 10.1128/mBio.02083-16 (PMC5312083; doi:10.1128/mBio.02083-16)
Supplement: FIG S4 [file mbo001173189sf4.pdf]

Figure S4

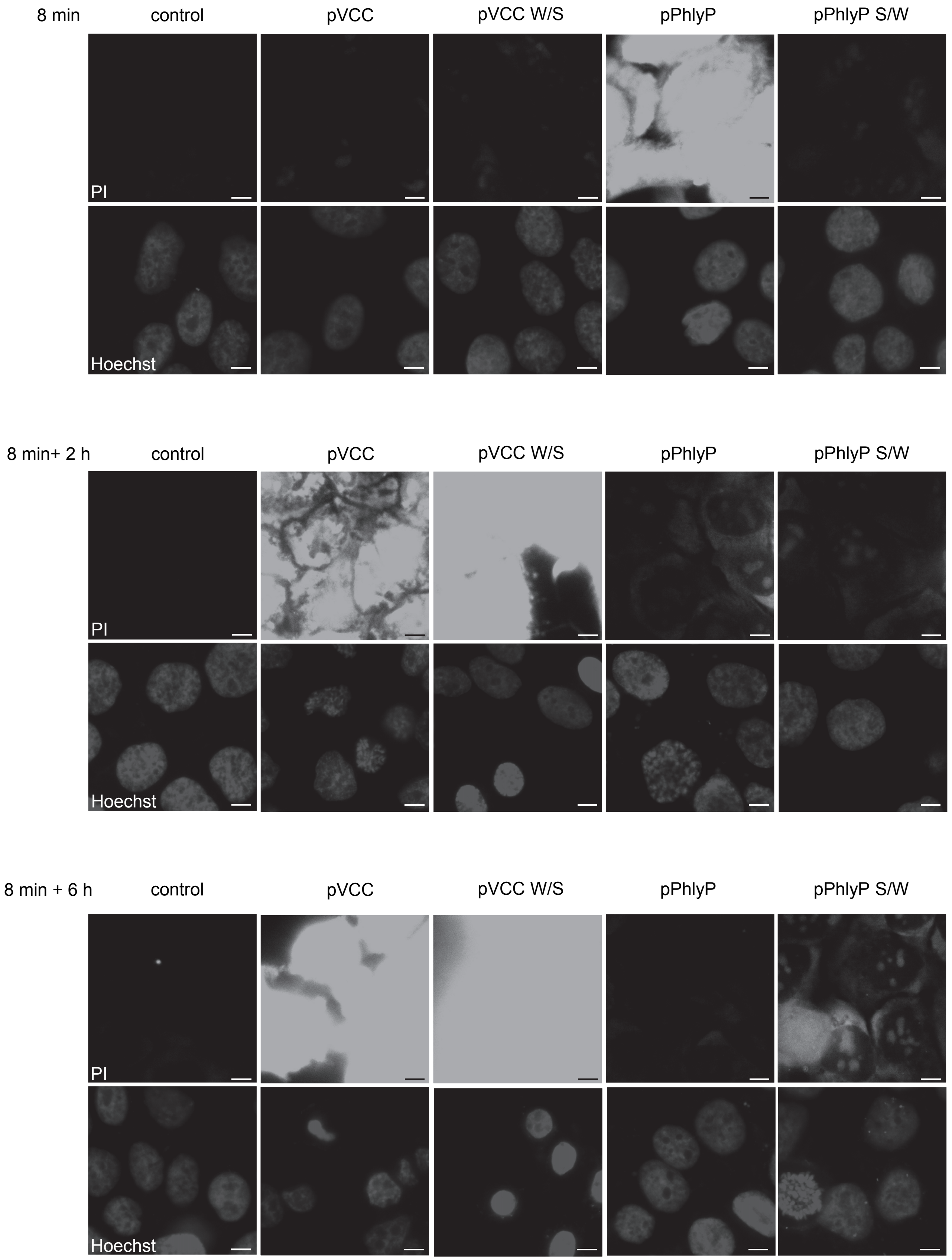

FIG S4 Single channel grey scale images corresponding to Figure 6A. HaCaT cells were incubated with pro-toxins (100 ng/ml). After 8min cells were washed and either immediately incubated with PI (50 µg/ml; 1 min; RT), or incubated for 2 h or 6 h before incubation with PI, fixation and microscopic examination.
