# Supplementary material for: Repair of a Bacterial Small β-Barrel Toxin Pore Depends on Channel Width
Source: mBio. 2017 Feb 14;8(1):e02083-16. doi: 10.1128/mBio.02083-16 (PMC5312083; doi:10.1128/mBio.02083-16)
Supplement: FIG S5 [file mbo001173189sf5.pdf]

Figure S5

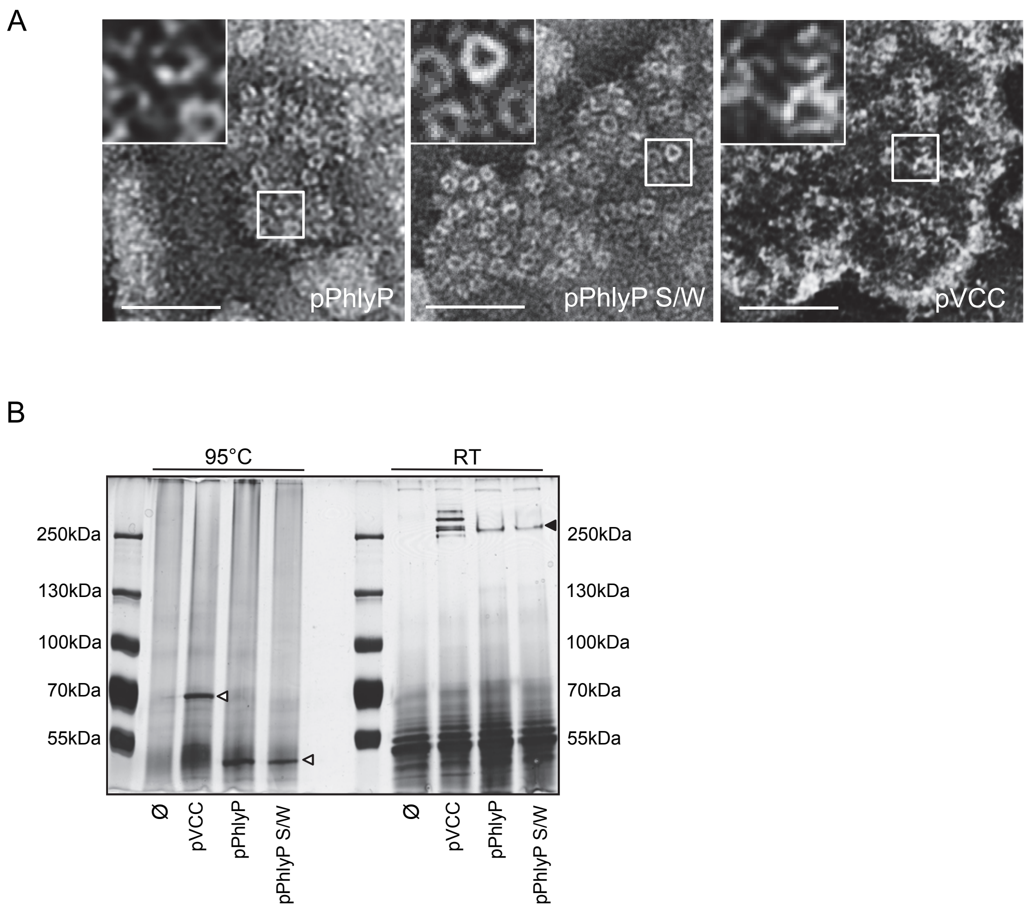

FIG S5 PhlyP S/W oligomerizes and forms annular structures. (A) Rabbit erythrocyte ghosts were loaded with pPhlyP, pPhlyP S/W or pVCC and analyzed by TEM. Scale bar = 50 nm. (B) Erythrocyte ghosts were incubated with pVCC, pPhlyP or pPhlyP S/W and separated by SDS-PAGE; and proteins were silver stained. The filled triangle indicates the presumed oligomers, the open triangles the monomers.
