# Supplementary material for: Repair of a Bacterial Small β-Barrel Toxin Pore Depends on Channel Width
Source: mBio. 2017 Feb 14;8(1):e02083-16. doi: 10.1128/mBio.02083-16 (PMC5312083; doi:10.1128/mBio.02083-16)
Supplement: FIG S6 [file mbo001173189sf6.pdf]

Figure S6

A

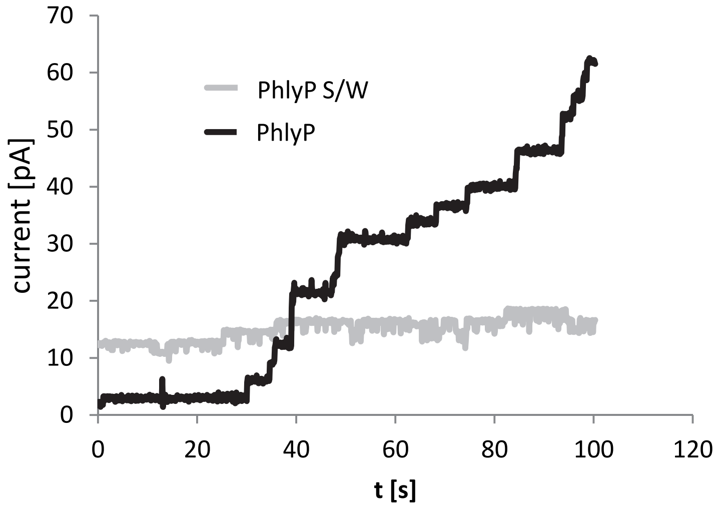

B

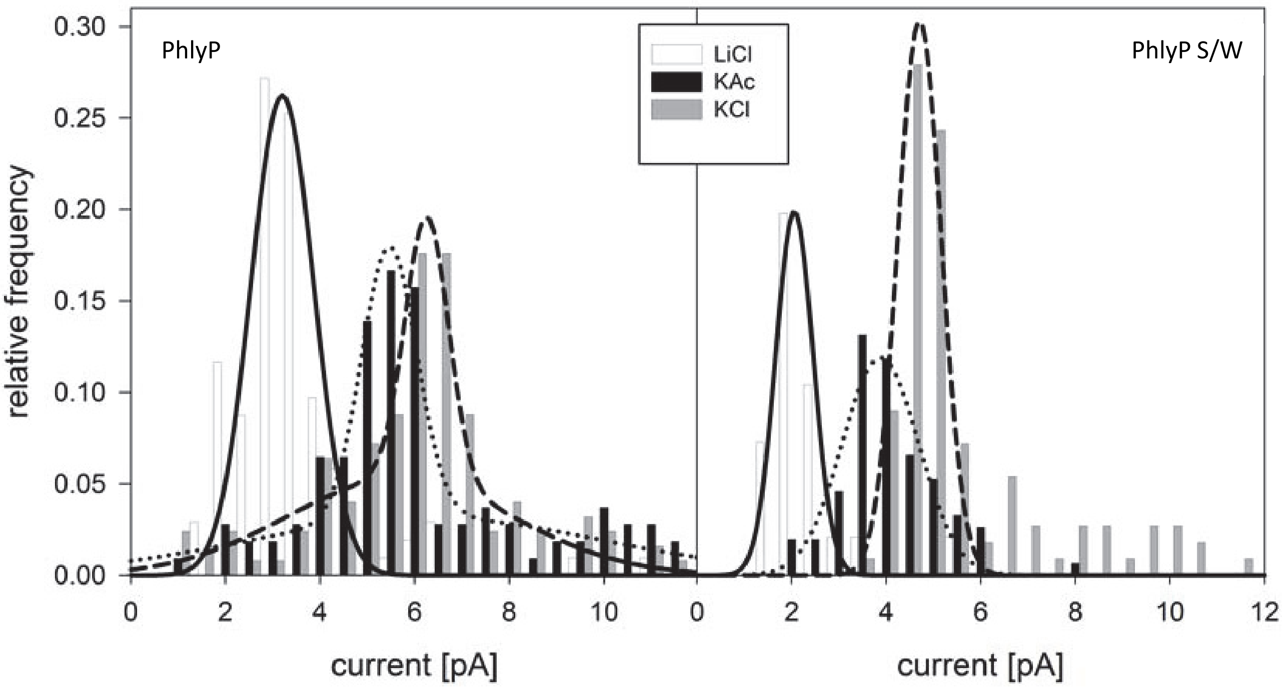

FIG S6 PhlyP pores are of rather high conductance and moderately cation-selective; conductance of PhlyP S/W pores is instable. (A) Single channel recordings on BLM were obtained with PhlyP or PhlyP S/W, samples of typical traces are shown; 100 mM Kac; overlay of raw data after filtering (15-point, 2nd order Savitzky-Golay-Filter). Note well-defined steps with only rare downward spikes with wild type PhlyP. In contrast, significant downward spiking and periods of reduced conductance were observable with PhlyP S/W; increasing toxin concentration led to rapid increase of the current to levels where individual steps could no longer be identified. (B) Multiple current traces from experiments similar to the ones shown in (A) were analyzed, and presented as histograms. Left: data for PhlyP, right: PhlyP S/W. White bars represent data obtained with 100 mM LiCl, black bars with 100 mM KAc and grey bars with 100 mM KCl. The distributions were analyzed based on one or two Gaussian functions. The relative frequency was obtained by normalizing to the total number of steps.
