# Supplementary figures and images for: Repair of a Bacterial Small β-Barrel Toxin Pore Depends on Channel Width
Source: mBio. 2017 Feb 14;8(1):e02083-16. doi: 10.1128/mBio.02083-16 (PMC5312083; doi:10.1128/mBio.02083-16)

Figure S7

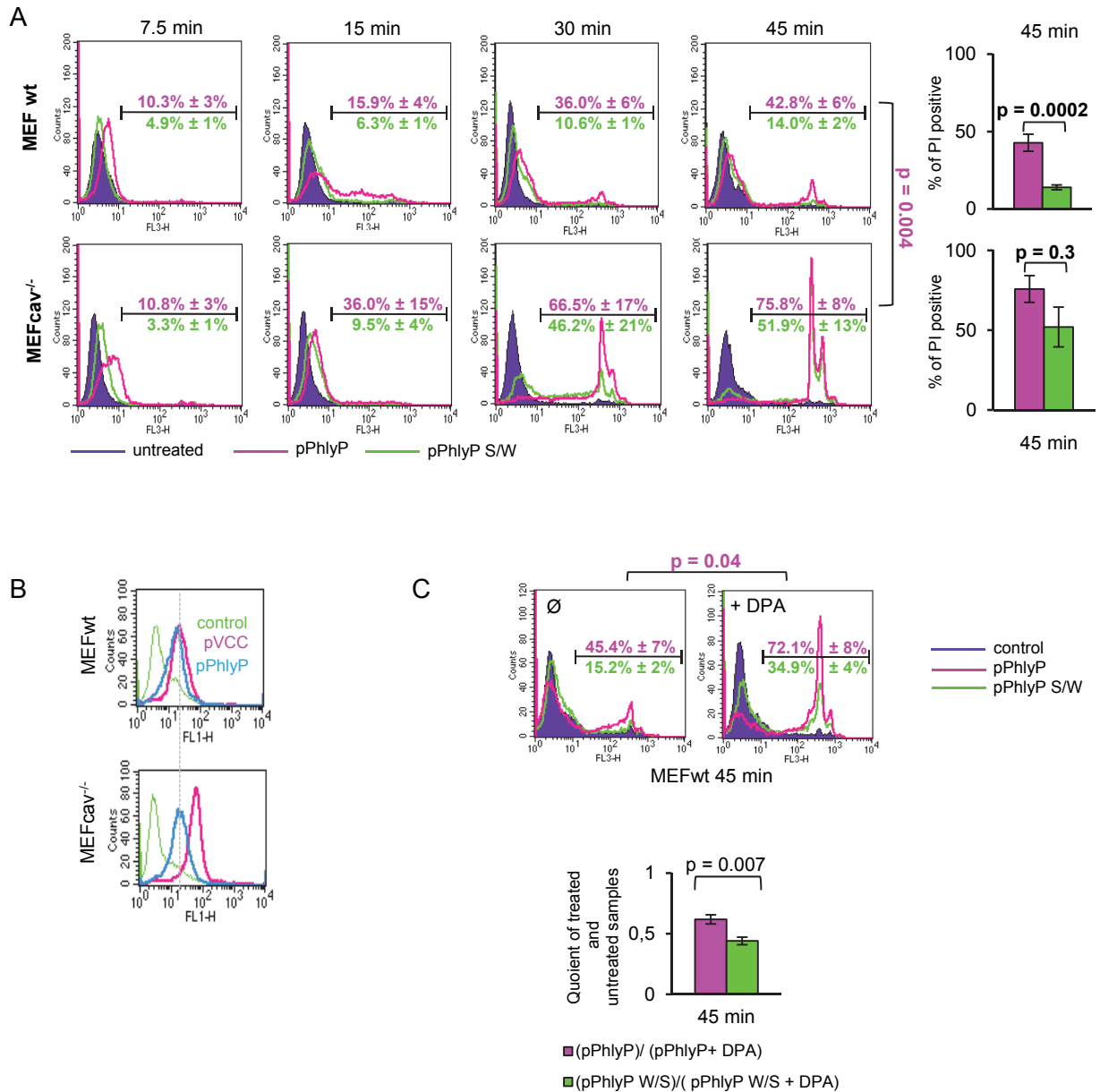

Supplement: FIG S7 [file mbo001173189sf7.pdf]
