# Supplementary material for: Repair of a Bacterial Small β-Barrel Toxin Pore Depends on Channel Width
Source: mBio. 2017 Feb 14;8(1):e02083-16. doi: 10.1128/mBio.02083-16 (PMC5312083; doi:10.1128/mBio.02083-16)
Supplement: TEXT S1 [file mbo001173189s1.docx]

**Supplemental Materials and Methods**

*Chemicals, antibodies*

SB203580 from Calbiochem, anti-(p) p38 Cell Signaling; anti-Ceramide (mAb MD 15B4) was from Enzo Life Sciences, Hoechst from Molecular Probes, EGTA from Roth , anti-LAMP-1 (1D4B) - AlexaFluor488, Fluo8-AM, PPADS and suramin-sodium were purchased from Santa Cruz, 4-methyl-umbelliferyl-N-acetyl-β-D-glucosaminide, blebbistatin, desipramin and PI were from Sigma. Affinity-purified rabbit polyclonal anti-peptide antibody specific for PhlyP (peptide sequence: CLWDGDKLKNSFEDK) was custom made by Genscript.

*Homology based modeling of the PhlyP pore*

*TAS* The sequence of PhlyP was modeled on the x-ray structure of VCC (3O44.pdb, ref. ([1](#_ENREF_1)). Alignment and structural modeling were performed by using MODELLER version 9.13 ([2](#_ENREF_2)). Alignment obtained with ClustalX ([3](#_ENREF_3)) yielded very similar results. The sevenfold symmetry was considered during the modeling process. Twenty models were generated, and ranked according to their root means square deviations (RMSD) of all atoms in the channel region, relative to the template 3O44.pdb. RMSD-values were calculated with CHIMERA ([4](#_ENREF_4)). Models with the highest and the lowest RMSD were selected for comparison with VCC channel structure. Overall quality of the models was assessed by calculating the Qmean score ([http://swissmodel.expasy.org/qmean/cgi](https://mail.uni-mainz.de/owa/redir.aspx?C=Fs23aHlrmUSJr-wpmSX8Hjkhf4tbddEIfpfTpGuhZWRYz9B-bp6qS4GrQn4GF7ByzigiW4z2UAA.&URL=http%3a%2f%2fswissmodel.expasy.org%2fqmean%2fcgi)) which can assume values between 0 and 1, with higher values reflecting higher reliability of the model. Qmean scores of models with highest and lowest RMSD for the channel region were 0.466 and 0.433, respectively.

*Site directed mutagenesis / primer*

Exchange of single aminoacids in pPhlyP S/W and pVCC W/S mutants was performed according to manufacturers instruction manual for QuikChange II XL Site-Directed Mutagenesis Kit (Agilent Technologies). In brief, mutations were introduced by PCR, using pTrcHisA-pPhlyP and pQE30-pVCC as templates, and primer pairs

5`-aactctataatcttgtgtattaaaagataaccattttgattgactataactagcactagcttg-3´ and

5`-caagctagtgctagttatagtcaatcaaaatggttatcttttaatacacaagattatagagtt-3´ or

5`-aagttatacccagagtcgcagcttaacctacaacacacaag-3´ and

5`-cttgtgtgttgtaggttaagctgcgactctgggtataactt-3´

for pPhlyP S/W and pVCC W/S mutants, respectively. PCR-products were digested with DpnI and transformed into XL10-Gold Ultracompetent cells. Mutated plasmid DNA was isolated from transformed clones and verified by custom DNA sequencing. For expression, pPhlyP S/W and pVCC W/S plasmid were transformed into NEB SHuffle Express competent *E. coli* (C3028)*.*

*Fluo-8-based* Ca^2+^*assay*

PFT-induced changes of [Ca^2+^]_i_ in HaCaT cells were monitored by using Fluo-8 from Santa Cruz Biotechnology Inc. in a TriStar LB 941 instrument from Berthold Technologies. HaCaT cells (30.000/well in 200 μl complete medium) were seeded in a 96-well format using black flat-bottom microplates (Greiner) and incubated at 37°C o/n. Cells were loaded for 30 min with a mixture of Fluo-8AM (100 μM), Cremophor 0.1% (w/v) and Probenecid 2 mM, toxin was automatically injected and fluorescence intensity recorded (5 s intervals, 3 min) in a TriStar LB 941 instrument from Berthold Technologies equipped with appropriate filters (center wavelengths excitation 485nm and emission 535nm); lamp intensity was set at 14000. In control experiments, supernatants of toxin-treated samples were consistently found to yield negative results for all time points, thus ensuring that increased fluorescence intensity in samples were not due to leakage of cleaved Fluo-8 ester from cells, but faithfully reported changes of [Ca^2+^]_i_.

*PI influx*

To assess influx of PI into cells, toxin-treated cells were washed using PBS at RT, incubated with PI (50 µg/ml) for 1 min, fixed PFA 2%, and nuclei were stained with Hoechst. Stained cells were washed again and coverslips were mounted. Samples were analyzed by wide field fluorescence microscopy using an Axiovert 200 microscope equipped with a Plan Apochromat 100 x / 1.4 aperture lens. Because toxin-treated MEF tended to detach from glass slides, PI influx in MEF was analyzed in suspension by flow cytometry using a FACScan Instrument (Becton Dickinson); 10.000 events were counted per condition. Cells were trypsinized, washed in PBS and stained as described above.

*Fluorescence microscopy*

Immunofluorescence staining for the lysosomal marker protein LAMP-1 or ceramide was performed with MEF, because we obtained unspecific staining with anti-human LAMP-1 specific antibodies and HaCaT cells. Antibody and staining protocol for LAMP-1 were as published ([5](#_ENREF_5)). Staining of MEF cells for ceramide was done with antibody 15B4 following a protocol described previously ([6](#_ENREF_6)). Cells were grown on glass coverslips o/n and incubated or not with ECPs as detailed in the figure legends. After incubation with first antibodies, cells were washed in PBS and subsequently fixed with 2% paraformaldehyde in PBS for 10 min at RT; and incubated with AlexaFluor®-conjugated secondary antibodies. Coverslips were mounted on slides with Fluoprep (bioMérieux® SA) and samples were examined in a Zeiss Axiovert 200M epifluorescence microscope equipped with a Plan Apochromat 100x/1.4 aperture lens. Digital images were acquired with a Zeiss Axiocam. Image processing was done using Zeiss AxioVision, Metamorph and Adobe Photoshop software.

*Western-blot*

Cells were lysed directly in loading buffer (10%(v/v) glycerol, 5%(v/v) 2-mercaptoethanol, 2%(w/v) SDS, and bromphenol blue) and heated for 5 min at 95°C. Proteins were separated by SDS-PAGE (10%) and electro-blotted onto nitrocellulose membrane. After blocking for 1 h at RT in skim milk in TBST, the membrane was incubated with a primary antibody, washed three times in TBST and incubated with HRP-conjugated second antibody for 1 h at RT. After three washing steps, bound antibody was detected by ECL, as descripted by the supplier (BM Luminescence Roche).

*FACS analysis of pPhlyP /pVCC-binding to MEF*

MEFwt or MEFcav^-/-^ cells were preincubated with metalloprotease inhibitors TAPI-0 and TAPI-2 (1 µM) for 45 min at 37°C. Subsequently, cells were trypsinized, washed and re-suspended in DMEM. After incubation with purified pVCC or pPhlyP for 1h on ice, cells were washed with ice cold PBS and fixed with 1% paraformaldehyde in PBS for 15 min on ice. Fixed cells were washed with PBS before they were incubated with mouse monoclonal primary anti-penta-His antibody (Qiagen). After 1 h at room temperature, cells were washed again with PBS and incubated with goat anti mouse- Alexa 488 conjugate (Molecular Probes) for 1 h at room temperature. Finally, cells were washed twice with PBS and re-suspended in PBS containing EDTA (1 mM) before FACS analysis.

*Transmission electron microscopy (TEM) of toxin-treated erythrocytes*

Toxin-treated erythrocyte ghost membranes were prepared; and TEM was performed as described in Rivas et al. 2015. In brief, RRCs (rabbit red cells) in osmoprotection buffer (20mM Tris-HCl, pH 7.0; 0.1% bovine serum albumin (BSA), 30mM dextran 4) were incubated with PhlyP (12 µg/ml), mutant PhlyP S/W (12 µg/ml) or pVCC (7.5 µg/ml) for 1h at RT. Subsequently RRCs were washed in osmoprotection buffer and lysed in 5 mM sodium phosphate, pH 8. RRC membranes were washed twice and then resuspended in 100 µl of PBS. Droplets of toxin-loaded ghost preparation were negatively stained with ammonium molybdate, and specimes were examined with a Zeiss EM 902 instrument.

*Conductance measurements*

Black lipid membranes were formed on chambers from Ionovation GmbH (Osnabrück, Germany). Typically, 0.4 μl of 5 mg/ml DPhyPC in decane were added to each side (about 1.4 ml per chamber). Bilayers were formed by moving the buffer solution up and down past the hole with a pipette, until the capacity increased to at least 27 pF. The stability of the bilayers was checked for 10 min before 1‐5 μl toxin (~10 nM final concentration) was added to the *cis*‐side (the *trans* side was on zero potential, the cis‐side set to 50 mV). Currents were monitored under voltage‐clamp conditions ([7](#_ENREF_7)) at 10 Hz sampling rate. Traces showed well developed steps (fig. S6). The steps were analyzed manually, only considering positive steps 0.5 pA amplitude.

*Endocytosis*

Fluid phase marker BSA-Alexa 488 (50 µg/ml) was applied to the culture medium of HaCaT cells; for microscopic analysis HaCaT cells were grown on glass coverslips. Next, cells were treated with PhlyP (75 ng/ml, 37°C, for different times) before either processing for microscopic analysis, or for FACs analysis.

*Exocytosis*

Exosomes were prepared as described in ([8](#_ENREF_8)). In brief, HaCaT cells, preincubated with DMSO, or 50 µM blebbistatin in DMSO for 30 min at 37°C were loaded with PhlyP (100 ng/ml), and incubated for 1 h at 37°C in the continuous presence of inhibitor/solvent. Subsequently, supernatants were collected and sequentially centrifuged at 300 x g, 1200 x g, 10000 x g and 100000 x g as described. Pellets were dissolved in SDS loading buffer and proteins were separated by 10% SDS-PAGE. Gels were analyzed by Western blot for the presence of PhlyP, flotillin, and caveolin-1.

**Supplemental references**

1. **De S, Olson R.** 2011. Crystal structure of the *Vibrio cholerae* cytolysin heptamer reveals common features among disparate pore-forming toxins. Proc Natl Acad Sci U S A **108:**7385-7390.

2. **Sali A, Blundell TL.** 1993. Comparative protein modelling by satisfaction of spatial restraints. J Mol Biol **234:**779-815.

3. **Larkin MA, Blackshields G, Brown NP, Chenna R, McGettigan PA, McWilliam H, Valentin F, Wallace IM, Wilm A, Lopez R, Thompson JD, Gibson TJ, Higgins DG.** 2007. Clustal W and Clustal X version 2.0. Bioinformatics **23:**2947-2948.

4. **Pettersen EF, Goddard TD, Huang CC, Couch GS, Greenblatt DM, Meng EC, Ferrin TE.** 2004. UCSF Chimera--a visualization system for exploratory research and analysis. J Comput Chem **25:**1605-1612.

5. **Qureshi OS, Paramasivam A, Yu JC, Murrell-Lagnado RD.** 2007. Regulation of P2X4 receptors by lysosomal targeting, glycan protection and exocytosis. J Cell Sci **120:**3838-3849.

6. **Grassmé H, Jendrossek V, Bock J, Riehle A, Gulbins E.** 2002. Ceramide-rich membrane rafts mediate CD40 clustering. J Immunol **168:**298-307.

7. **Chakraborty T, Schmid A, Notermans S, Benz R.** 1990. Aerolysin of Aeromonas sobria: evidence for formation of ion-permeable channels and comparison with alpha-toxin of Staphylococcus aureus. Infect Immun **58:**2127-2132.

8. **Husmann M, Beckmann E, Boller K, Kloft N, Tenzer S, Bobkiewicz W, Neukirch C, Bayley H, Bhakdi S.** 2009. Elimination of a bacterial pore-forming toxin by sequential endocytosis and exocytosis. FEBS Lett **583:**337-344.
